# Structural conservation among the rhodopsin-like and other G protein-coupled receptors

**DOI:** 10.1038/srep09176

**Published:** 2015-03-17

**Authors:** Mikitaka Kinoshita, Tetsuji Okada

**Affiliations:** 1 Department of Life Science, Gakushuin University, 1-5-1 Mejiro, Toshima-ku, Tokyo 171-8588, Japan

## Abstract

Intramolecular remote coupling within the polypeptide backbones of membrane proteins is difficult to analyze owing to the limited structural information available at the atomic level. Nonetheless, recent progress in the crystallographic study of G protein-coupled receptors (GPCRs) has provided an unprecedented opportunity for understanding the sophisticated architecture of heptahelical transmembrane (7TM) bundles. These 7TM bundles can respond to a wide range of extracellular stimuli while retaining the common function of binding trimeric G proteins. Here we have systematically analyzed select sets of inactive-like 7TM bundles to highlight the structural conservation of the receptors, in terms of intramolecular C_α_-C_α_ distances. Distances with the highest scores were found to be dominated by the intrahelical distances of helix III, regardless of the choice of bundles in the set, indicating that the intracellular half of this helix is highly conserved. Unexpectedly, the distances between the cytoplasmic side of helix I and the extracellular region of helix VI provided the largest contribution to the high score populations among the interhelical pairs in most of the selected sets, including class B, C and frizzled receptors. These findings are expected to be valuable in further studies of GPCRs with unknown structure and of other protein families.

Almost all eukaryotic organisms utilize G protein-coupled receptors (GPCRs) for sensing both external and internal signals, such as those from chemical substances, hormones, and photons^1^,^2^. Recent advances in the structural study on the receptors of this superfamily have provided a wealth of information, promoting a wide range of researches, including for example, the rational design of drugs with improved efficacy and specificity^3^,^4^. However, a substantial number of unexplored targets found from unicellular eukaryotes to humans remain to be investigated in atomic detail.

A set of coordinates for a given family of proteins can be utilized to develop reliable methods for predicting the structure of related proteins, and to gain insights into the mechanisms underlying molecular evolution. There are few membrane protein families suitable for conducting detailed analysis using a wealth of data with preferable variations in both the structure and the sequence. The transmembrane domains of GPCRs, accommodating both the functionally common and the variable parts within a bundle of seven helical segments confined in the lipid bilayer, offer an excellent template model in terms of the biological significance of their structure/activity relationship and the moderate structural and sequence variations.

Previous studies on the arrangement of the seven transmembrane (7TM) helices of the inactive-like crystal structures of GPCRs have confirmed that the positions near to the highly conserved residues in the rhodopsin family, designated as *.50 (asterisk indicates helix number) with Ballesteros & Weinstein (BW) numbering^5^, exhibit relatively small positional deviations among receptors^6^. However, the degree of conservation in terms of the intramolecular distances has not yet been quantitatively examined. RMSD values, often used for evaluating similarity between a pair of receptors, are not suitable for determining whether any common features are conserved among a set of receptors. Furthermore, recently determined structures of non-rhodopsin family GPCRs have demonstrated that the apparently important helix VI of the 7TM bundles deviates significantly from that of the rhodopsin family receptors^7^,^8^,^9^. This finding has led to questions regarding the types of structural conditions that are essential to maintaining a common function.

To address these issues, we performed an extensive distance analysis that included calculation of all the pairwise intramolecular distances between the C_α_ atoms within 200 residue 7TM bundles. Conservation was scored according to the coefficient of variation calculated for the receptors selected in a given set. By testing various sets of receptor combinations, we found that this simple method of analysis provided valuable insights into the architecture of the GPCR superfamily.

## Results

### Receptor selection

The 7TM bundles, each containing 200 residues, were defined as previously reported for rhodopsin family receptors^6^. Thus, 200 × 199/2 = 19,900 values were examined for each of the bundles. Initially we analyzed 59 inactive bundles that were available until July 2013 (set 0) that had a crystallographic resolution higher than 3.6 Å (supporting table S1). The results obtained from this set led us to perform a further detailed study, and the list of the structures examined is summarized in supporting table S2. For instance, set 1 was composed of the structures of the rhodopsin family that were available until January 2014, and that had a resolution higher than 3.3 Å. Thus, set 1 contained 6 rhodopsin (4 bovine, 2 squid), 10 adrenergic (5 each for β_1_ and β_2_), 5 A_2A_ adenosine, 7 chemokine (5 CXCR4, 2 CCR5), 4 other amine (2 dopamine, 1 histamine, 1 muscarinic), 6 opioid (2 kappa, 2 N/OFQ, 1 delta, 1 mu), 1 sphingosine and 1 thrombin receptor bundles. In this set, the total number of bundles was decreased to 40, reducing possible bias toward the adrenergic receptors for which the greatest number of crystal structures is available. To evaluate the findings obtained for the rhodopsin family receptors, set 2 was defined as set 1 with the addition of three non-rhodopsin family bundles, one class B CRF_1_ receptor and two chains of smoothened receptor. From set 2, the five subfamilies- rhodopsin, adrenergic, adenosine, chemokine, and opioid receptors were removed to form set 3, 4, 5, 6, and 7, respectively, to see whether any subfamily specific features exist. In sets 8 and 9, a maximum of three bundles or one bundle, respectively, for each of the receptors was included, to reduce any possible bias from larger contribution from rhodopsin family receptors. Thus, set 9 contained 18 unique receptor bundles; however this set was still biased toward the rhodopsin family, as it contained 16 rhodopsin family members and 2 non-rhodopsin family members. This was simply due to the current limited number of available structures for non-rhodopsin family receptors. Recently reported class C receptor^10^ was also taken into account in set 10, which contained set 9 and mGluR1 receptor.

Since our current analysis focused only on extracting information on structural features that are relatively invariable among subfamilies of receptors that vary from each other by at least more than 1.5 Å in the overall RMSD for the TM region, any possible effects of various modifications (that mostly result in changes of < 1 Å in overall RMSD) applied to each receptor (Table S2) did not appear to affect the results significantly. As shown below, this is confirmed by the fact that we could detect a subtype–specific feature in adrenergic receptors for which both modified and unmodified structures were included in the analysis.

### Ranking procedure

For each of the C_α_-C_α_ pair distances in the sets, the average, maximum, minimum, and standard deviations were calculated. As a simple and rational measure (score) of the conservation among the bundles, we chose the inverse of coefficient of variation, wherein this coefficient is the average divided by the standard deviation. The scores obtained in the current study varied from ~ 1.5 to ~ 250. With this scoring, the higher numbers (ranks with a smaller number) were dominated by C_α_ pairs of adjacent amino acid positions, and these were omitted from the ranking because they do not appear to provide much information on structural conservation.

**Figure 1:**
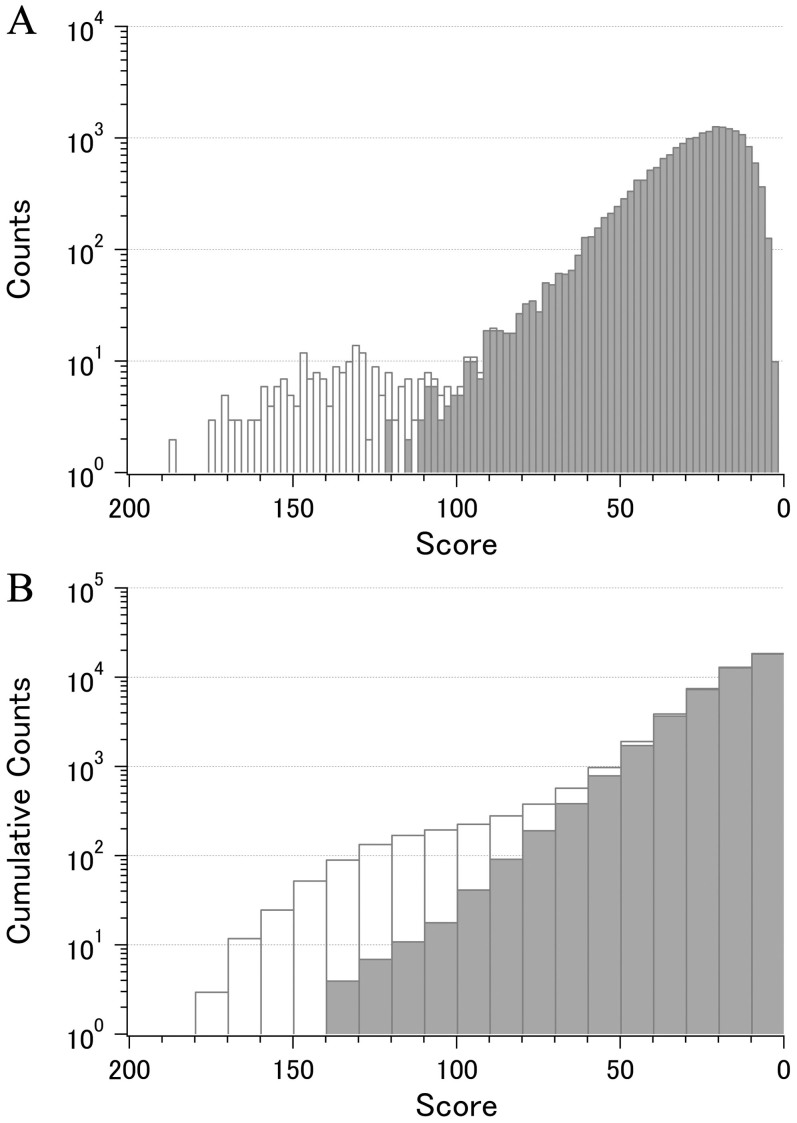
Score distribution of 19,900 C_α_- C_α_ pairs. Calculated for set 1 containing forty 7TM bundles of rhodopsin family receptors with (blank bars) or without (filled bars) the contribution of 193 adjacent pairs. A. Counts in each score range. B. Cumulative counts.

Figure 1 shows the distribution of 19,900 C_α_ pairs in set 1 over the whole scoring range with or without the contribution of 193 adjacent C_α_ pairs. The cumulative count plot (Fig. 1B) indicates that C_α_ pairs found in the top 1,000 ranks have scores greater than approximately 50, which corresponds to a 0.4 Å standard deviation for the average distance of 20 Å, and is fairly higher than the average score of 29.4 obtained for all 19,900 pairs. Since the top 1,000 pairs amounted to only 5% of the total, detailed analysis of this population is a reasonable approach for examining the intramolecular distance conservation among the receptors in a given set.

### Data statistics

**Figure 2:**
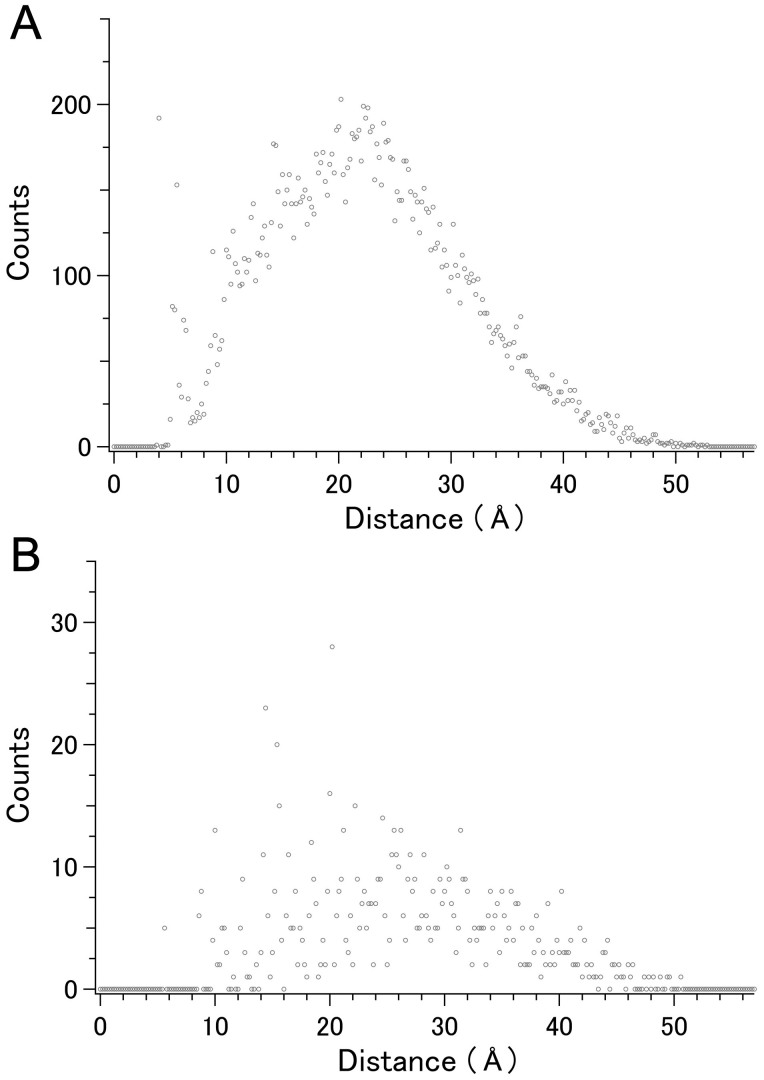
Average distance distribution of C_α_- C_α_ pairs in set 1. A. 19,900 pairs including the contribution of 193 adjacent pairs. B. The top-ranked 1,000 pairs without the contribution of 193 adjacent pairs. The point for 1000th rank included in panel B corresponds to rank 1193 in panel A, and a point corresponding to 193 adjacent pairs at a distance of ~ 3.8 Å, identical to that shown in panel A, has been omitted from panel B to make its vertical scale appropriate.

We next investigated whether the population of higher scoring C_α_ pairs was biased toward either long or short distances. Figure 2a shows the distribution of the average distances for the 19,900 C_α_ pairs in set 1. The maximum value of around 21 Å includes, for example, intrahelical distances separated by four pitches. Comparison of the average distance distribution for the C_α_ pairs ranked within the top 1,000 score with this distribution showed that both were similar (Fig. 2B), indicating that the current scoring was not biased toward short or large distance populations.

**Figure 3:**
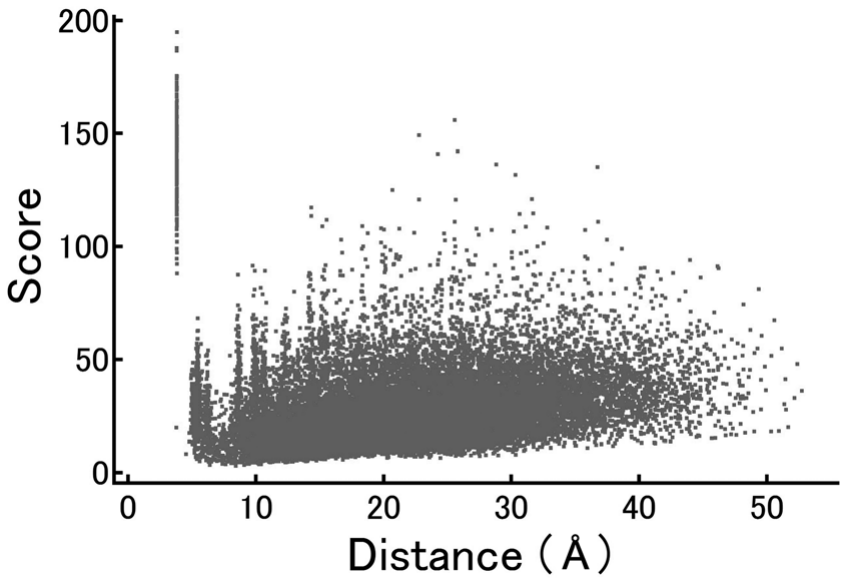
Correlation between score and the average distance of the 19,900 C_α_- C_α_ pairs in set 1.

The relationship between the distance and the score was further examined using a scatter plot of the 19,900 C_α_ pairs (Fig. 3) of set 1. As described earlier, the population with scores ranging from 90 to 200 at the distance of approximately 3.8 Å originates from adjacent pairs of amino acids. The remainder of the distribution appeared uniform across the whole range of distances. While the lower limit of the scores tended to increase slightly at longer distances, our detailed analysis focused only the scores higher than approximately 50. Therefore, these statistics support the assumption that the present simple scoring system would be useful in extracting the conserved arrangement of backbone among the receptors.

### Evaluation of high rank pairs

**Figure 4:**
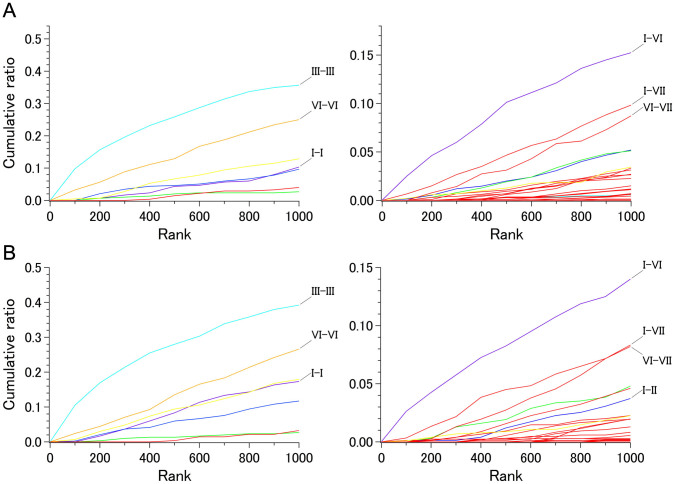
Cumulative ratio of the number of C_α_- C_α_ pairs in the top-ranked 1,000. A. Set 0 containing 59 rhodopsin family 7TM bundles. B. Set 1 containing 40 rhodopsin family 7TM bundles. Left: intrahelical pairs, Right: interhelical pairs. Intrahelical pairs are colored as follows; purple, helix I; blue, helix II; cyan, helix III; green, helix IV; yellow, helix V; orange, helix VI; red, helix VII. Interhelical pairs are colored as follows; blue, I–II; cyan, I–III; green, I–IV; purple, I–VI; yellow, III–V; red, other pairs.

We classified the top 1,000 ranking C_α_ pairs into 28 helix pairs (7 intrahelical and 21 interhelical) based on the helices to which the C_α_ belonged. Because the total number of C_α_ pairs differs among the 28 helix pairs depending on the length (number of amino acids) of each helix, the C_α_-C_α_ counts in the top 1,000 were compared among the 28 helix pairs after normalization. For instance, the total number of intrahelical C_α_-C_α_ distances of helix III was 581 since we had selected its TM region from 3.22 to 3.55 (34 residues). Therefore, the 220 C_α_-C_α_ distances found in the top 1,000 ranked pairs of set 1 corresponded to 37.9% of the total (see below and Fig. 4B). In the same way, we obtained the patterns of contribution from each of the 28 helix pairs to the top 1,000 ranked pairs for the 11 sets (from set 0 to set 10).

**Figure 5:**
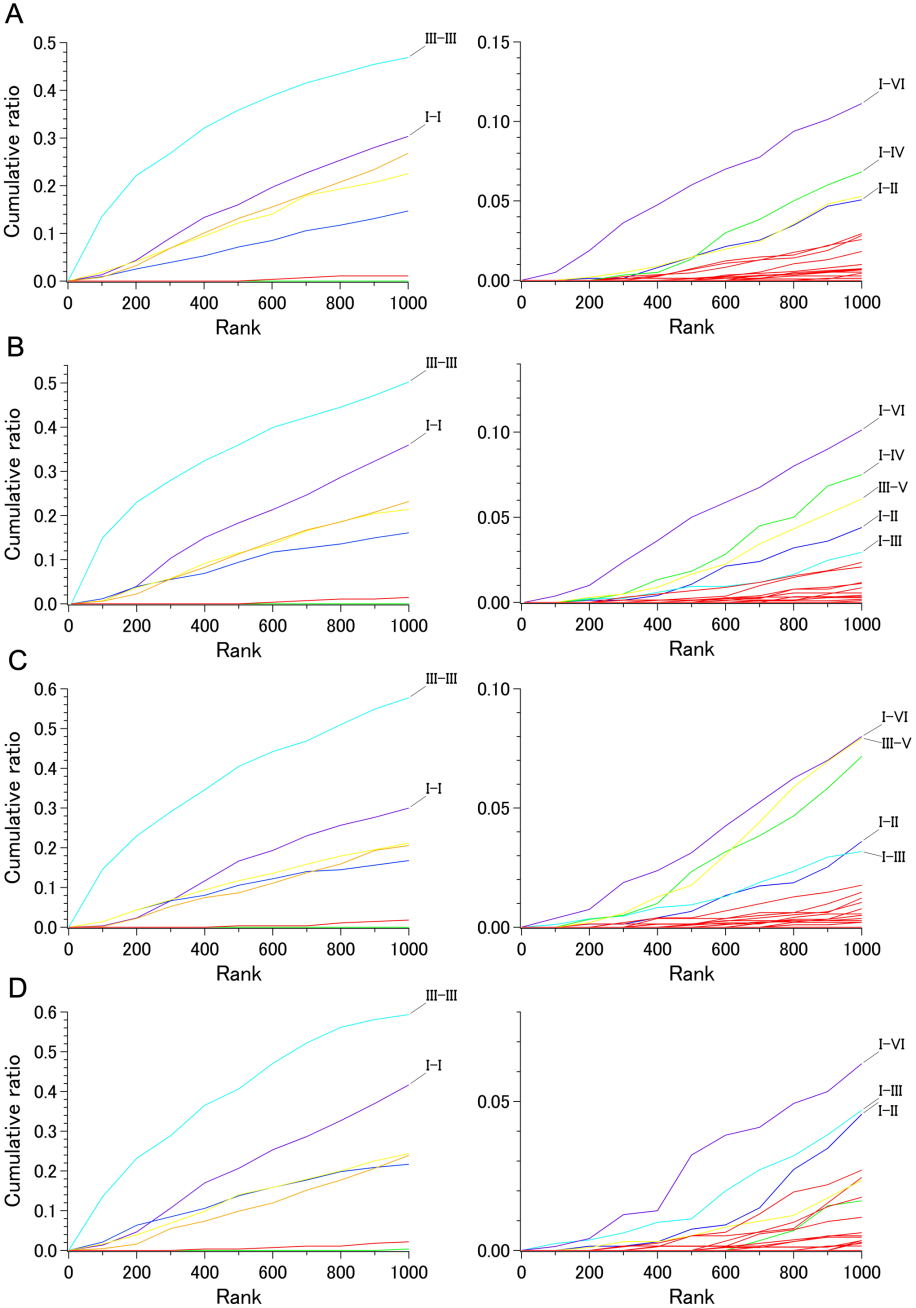
Cumulative ratio of the number of C_α_- C_α_ pairs in the top-ranked 1,000. A. Set 2 containing 40 rhodopsin family 7TM bundles and three non-rhodopsin family bundles. B. Set 8 containing 31 rhodopsin family 7TM bundles and three non-rhodopsin family bundles. C. Set 9 containing 16 rhodopsin family 7TM bundles and two non-rhodopsin family bundles. D. Set 10 containing 16 rhodopsin family 7TM bundles and three non-rhodopsin family bundles. Left: intrahelical pairs, Right: interhelical pairs. Coloring of the plots is the same as in figure 4.

Figure 4 shows the cumulative numbers (ratios to the total) of the C_α_ pairs found in the top 1,000 ranks for 28 helix pairs for sets 0 and 1, both of which contain only rhodopsin family bundles. The corresponding data containing non-rhodopsin receptors are shown in figure 5 (sets 2, 8, 9, and 10, the latter of which had a smaller proportion of rhodopsin family members) and in supporting figure S1 (sets 3 to 7, for each of which a specific receptor subfamily was omitted from set 2).

The higher ranked C_α_-C_α_ distances in all the sets were mainly from the intrahelical part of helix III, regardless of the inclusion of non-rhodopsin family receptors (Fig. 4, 5, Fig. S1). In the extreme cases of set 5 (with no adenosine receptors), set 9 (bundles representing 18 unique receptors), and set 10 (set 9 plus class C receptor), nearly 60% (approximately 335 of 561) of the possible C_α_ pairs in this helix were ranked in the top 1,000. These observations quantitatively demonstrate that the overall arrangement of this helix is much more conserved than that of the other helices among all the GPCRs whose structure is known. A slight increase in the higher ranked C_α_ pairs from helix III in set 5 is likely due to the uniquely distorted character of this helix in the adenosine A_2A_ receptor, as has been documented previously^11^.

Next we examined the conservation pattern in more details, according to the previously described horizontal sectioning of the 7TM bundle of 200 residues^6^ (Table 1). As shown in Fig. S2, we defined five sections to each of the helices. Thus, the possible number of intersection pairs is 630 (105 intrahelical and 525 interhelical), for the 7TM bundle. For each of these pairs, the total number of possible C_α_-C_α_ combination varied from 9 (section 1 of helix I and section 5 of helix 7, each of which contains only three residues) to 90 (section 1 of helices VI and VII, each of which contains 10 and 9 residues, respectively). Therefore, we evaluated the distance conservation for each of the intersection pairs by calculating the ratio of the number of C_α_ pairs found in the top ranked 1,000 to the total number of possible pairs (ratio column in Table 1).

Within helix III, our data show that the intracellular side appears to be more structurally conserved than the intracellular side. Direct evidence for this can be seen when comparison of the values in Table 1 is made between section pairs 2–3 and 3–4. A higher number of pairs from the 3–4 section are ranked in the top 1000, indicating that conservation of the distance from the center of helix III is more pronounced on the intracellular side. Importantly, the results of this comparison would not be affected by how the residue range for this helix was chosen; for example, a shorter length of this helix, excluding a few residues from both the intracellular and extracellular termini, should provide the same result.

Figure 4 also demonstrates that helices IV and VII are the most variable in the rhodopsin family; this is even more apparent when the non-rhodopsin family receptors are included (Fig 5 and Fig S1). Furthermore, the results of our analysis suggest that helical segments judged as regular α-helices by DSSP^12^ in many of the receptors (helices I, III, and VI) tend to exhibit high intrahelical distance conservation.

### Interhelical distance conservation

With regard to the interhelical components, a significant number of C_α_-C_α_ distances of the I–VI helix pair were found in the higher ranked population of sets 0 and 1 (Fig. 4). This finding was rather unexpected because no direct contact could be found between any parts of these two helices. The positions on helix I in the higher ranked I–VI pairs were found mostly in sections 4 and 5, a region that spans from around the conserved asparagine (BW no. 1.50) to the cytoplasmic end. On the other hand, positions on helix VI involved in the higher ranked I–VI pairs were found in sections 1 and 2, extending from the extracellular end towards the region near the conserved proline (BW no. 6.50).

Importantly, the conservation of distances between the cytoplasmic side of helix I and the extracellular side of helix VI was also apparent in all of the sets containing non-rhodopsin family receptors (Fig. 5 and Fig. S1). This finding was surprising as the superimposition of either the class B CRF_1_ receptor or smoothened receptor with the rhodopsin family receptors indicated a significant displacement of the extracellular side of helix VI, while the cytoplasmic side of helix I matched rather well. These observations might indicate that the deviation at the extracellular side of helix VI between the rhodopsin family and other receptor families reflects the presence of some directional restraint within a 7TM bundle, resulting in a relatively conserved distance from the cytoplasmic part of helix I.

Another interesting observation was the outstanding conservation of the distances between helices III and V in some of the sets containing non-rhodopsin family receptors (Fig. 5 and Fig. S1). Although this feature was not evident in set 10 where class C receptor was included (Fig. 5D), detailed examination indicated that there were still some high score pairs between the cytoplasmic side of helix III and the extracellular part of helix V.

We also noticed that a significant increase in population in the higher-ranked interhelical distances of the non-rhodopsin family-containing sets was attributable to the pairs between helices I and IV (Fig. 5). Furthermore, I–III and I–II helix pairs contributed significantly to the higher-ranked interhelical distances in the case of set 10. These observations and the intrahelical distance conservation data shown above suggest that positioning of helix I within the 7TM bundle is substantially conserved among all GPCRs.

**Figure 6:**
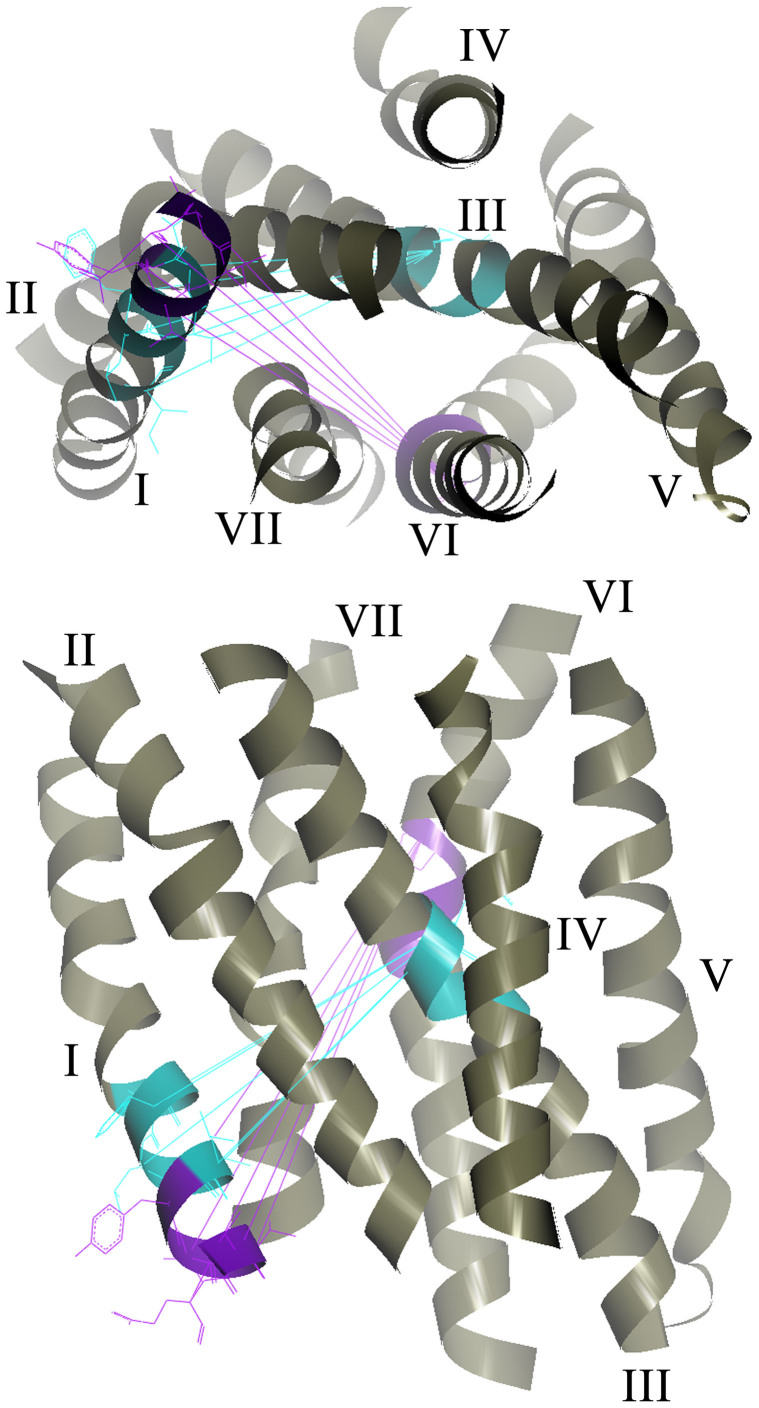
Graphical representation of conserved interhelical distances in set 10 including 19 unique bundles from all GPCRs with known structure. Upper: top view from the cytoplasmic side. Lower: side view from helices II and IV. Interhelical section pairs of conserved distance are colored on the 200 residue 7TM backbone of bovine rhodopsin template (1U19-A) as follows; purple, I–VI; cyan, I–III. All of the lines shown between the pairs have scores of more than 50. For clarity, the pairs involving the residues of BW no. 3.37 and 6.50 are shown by these lines.

**Figure 7:**
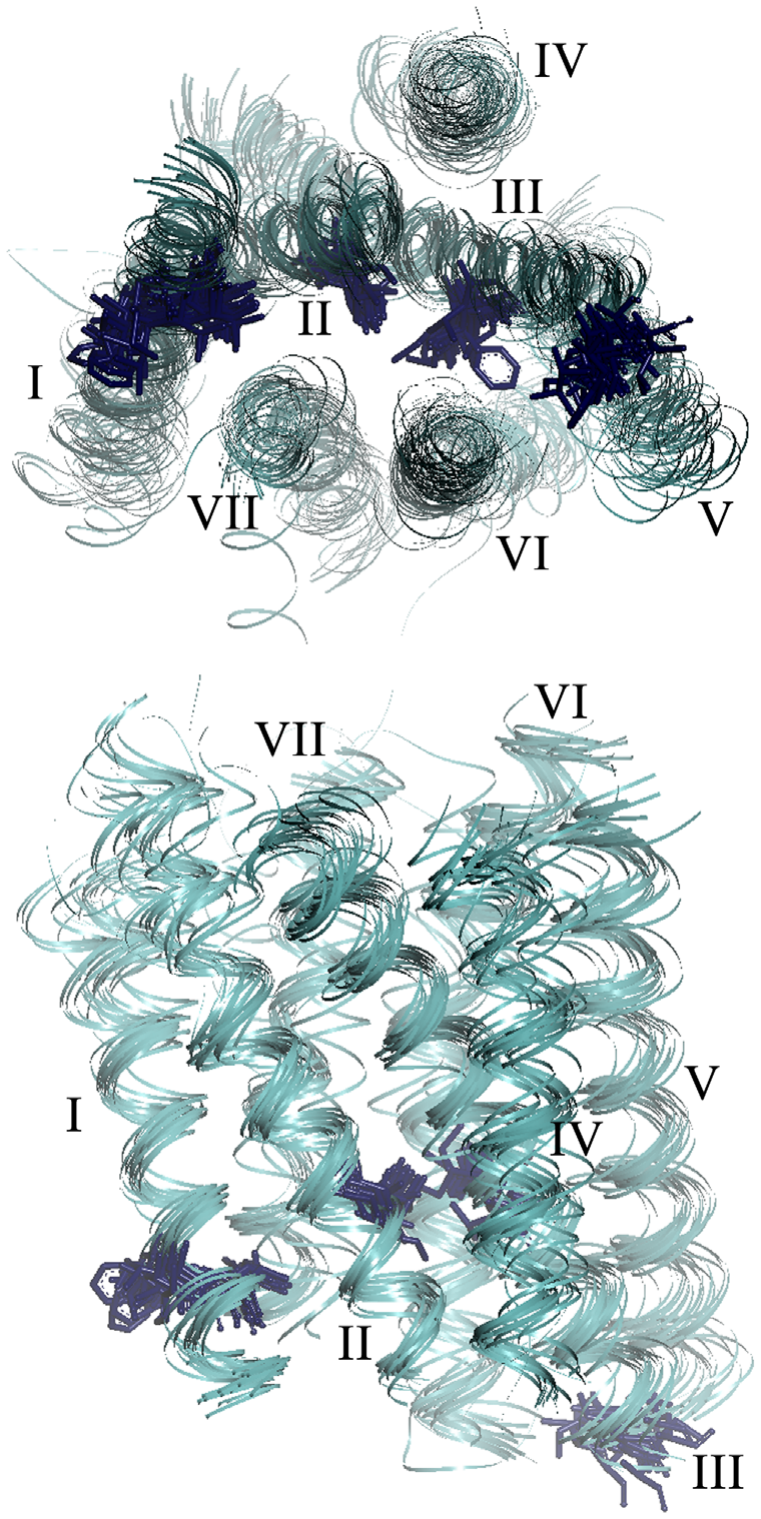
Graphical representation of conserved residues among 19 receptors in set 10. Upper: top view from the cytoplasmic side. Lower: side view from helices II and IV. Five conserved positions in helices I, II, and III (Fig. S3) are shown as sticks on each of the 7TM backbone ribbons. These residues correspond to BW no. of 1.52, 1.53, 2.46, 3.43, and 3.54.

The distance conservation results are summarized graphically in Figure 6, which includes lines connecting the 5 higher-ranked C_α_-C_α_s in each of the major interhelical section pairs in set 10. All of these C_α_ pairs had scores higher than 50. Figure 7 shows the positions of residues conserved among the receptors in set 10. In summary, the present results are consistent with the previous observation that positional deviations among rhodopsin-family receptors are small on the intracellular side of helices I, II, III, and in the middle of helix VI,^6^ and suggest that interhelical distance conservation correlate well with the presence of conserved amino acid type at some specific positions in the GPCRs with known structure. While no single position in the TM bundle is 100% identical among the receptors examined (Fig. S3), our analysis suggests that four of the five conserved residues, all of which are in section 4 (the middle–to–intracellular region), contribute to determining the basic arrangement of the bundle, at least from helices I to III, mostly by hydrophobic interactions. This interpretation explains why the relative contribution of the I–II and I–III pairs to the high score population increases as the ratio of the non-rhodopsin-family chains increases in a set (Fig. 5).

## Discussion

Statistical and quantitative analysis of the sequences and the three-dimensional structures of a protein family provide insights into functionally relevant and evolutionary significant regions. In the case of GPCRs, one of the most intriguing questions is how the common 7TM bundle core works for diverse members that convert a variety of signals, mostly captured at the middle to the extracellular part of the bundle, to the degree of coupling with the cognate heterotrimeric G proteins at the cytoplasmic surface. Although the basic folding pattern is similar for the seven helices in all the receptors of known structure, it is now increasingly evident that there are significant variations in spacing and shape of the helices depending on the subfamilies they belong.

Structural conservation and deviation in the 7TM region of GPCRs has been examined mostly based on superimposition and RMSD examination, and has shown little ambiguity as long as the receptors belonged to the rhodopsin family. The C_α_ positions at or near to the conserved residues (*.50 in BW numbering) exhibited relatively small deviations among the receptors^6^. However, recent determination of non-rhodopsin family receptor structures presents challenges concerning the reliable comparison and quantitation of differences among the 7TM regions. In fact, superimposition could vary, depending on how it was performed, and RMSD analysis does not appear to provide any quantitative results, as the present study does, such as the remarkable conservation of the structure of helix III and the I–VI, I–III distances.

Furthermore, rational interpretation of the increasing data on the relation between the type of bound ligand and the degree of conformational change requires understanding of common structural features that define the functional basis of this large family of membrane proteins. Here, we demonstrate that intramolecular distance conservation scoring provides a reasonable measure of evolutionary conserved basic architecture of GPCRs. The present results on distance conservation are consistent with a separate examination of the backbone torsion angles (phi, psi) of set 10: the lowest deviations are found in the sections I–4 (5.2° and 6.9° for phi and psi, respectively), III–4 (5.4° and 8.2°), and VI–2 (5.1° and 7.8°).

Although the amount of available structural data is limited, our results clearly show which part of the seven helices is the most or the least conserved in terms of the intra and interhelical distances (Fig. 5). Practically, such information should be useful in screening from a set of automatically constructed model for a receptor of unknown structure. Additionally, identification of conservation patterns will further evoke studies from evolutionary point of view. For instance, it would be interesting to analyze microbial 7TM retinal proteins with the current scoring method (see below).

The most conserved intrahelical distances are assigned at the spacing containing five turns approximately from 3.32 to 3.50. In fact, close examination of this part shows no significant distortion for all the receptors of known structure. Therefore, our result suggests that the regular and conserved shape of this helical segment is a key to the common functionality of GPCRs. Interestingly, the distances between the extracellular part of this segment and the cytoplasmic part of helix I is also conserved (Fig. 6). These findings, in conjunction with the intrahelical conservation of helix I and inter-helical conservation between the cytoplasmic part of helix I and the part around 6.50 of helix VI, highlight previously unidentified intramolecular spacing that might form the critical conditions for the functionality of GPCRs. Since the two regions described for helices III and VI affect the level of activity, it is conceivable that their relative position to a distant point (e.g., the cytoplasmic part of helix I) appears to be conserved.

Distance matrix analysis has been used for detecting the conformational transitions between two states within a protein^13^. The recent high-resolution structural models of GPCRs are valuable sources of data for extracting structural information regarding the conservation and variation among the evolutionally remote members of protein families, by systematically analyzing the intramolecular distances. To our knowledge, the variety or the quantity of structural data required for this sort of analysis are not available for other membrane protein families. The structures of a related family of 7TM proteins, the microbial retinal proteins, have been extensively investigated^14^. While the sequence variation of these proteins is limited, application of the current approach to this family of proteins appears to demonstrate distinct features from GPCRs in the intramolecular conservation patterns (unpublished data).

The statistical analysis presented makes use of only one snapshot of these inherently dynamic and fluctuating molecules^15^. Therefore, the quantity and the quality of available structural data would be expected to significantly affect the reliability of the inferred results. Indeed, the present scoring and ranking were reasonably sensitive to the choice of structures in a given set, especially with regard to interhelical pairs. It is possible that further structural data would improve the characterization of each of the families within the whole GPCR superfamily.

Changes in the intramolecular interatomic distance of specific pairs of amino acids have been frequently examined when estimating the trajectories for molecular dynamics simulations^16^,^17^. As a reference, our 50 ns simulation of the delta opioid receptor indicated that the scores calculated for the trajectory of the distances between the C_α_s of BW numbers 1.59 and 6.50, one of the highest-ranked-pair in the present study (score approximately 87 in set 10), was 51. On the other hand, the corresponding score between 3.50 and 6.30, which was expected to be sensitive to the state of receptor activity, was 12, which was comparable to that obtained in set 10 (score 5.6).

The present study focused only on the inactive-like structures that were obtained in the presence of antagonists, inverse-agonists or negative allosteric modulator. However, the degree of conformational “inactiveness” might differ among the structures in a given set depending on the type of ligand bound to each of the receptors. Therefore, the higher-ranked section pairs described may also be structurally conserved in the active-like states. While a direct comparison between the results obtained from inactive-like and active-like sets would be difficult due to the limited variety of receptors in the active-like set, further analysis of the active-like set is likely to provide valuable insights into the activation mechanism of the rhodopsin family receptors.

Analysis of the conserved interhelical pairs in each set appears to provide subfamily-specific information. In the case of set 4, where adrenergic receptors were omitted, we observe an increase in high score contribution from the III–V pairs. This result is consistent with the fact that superimposition of the bundles clearly showed that helix V of the β_1_ and β_2_ adrenergic receptors was shifted slightly upward (toward the extracellular side) from the other receptors while helix III did not exhibit any features among them.

On the other hand, deviations can also be accommodated without changing the interhelical distances. Previously reported significant deviations of bovine rhodopsin from other receptors with respect to the positioning of the extracellular part of helix III^6^ did not appear to affect its distance to the cytoplasmic end of helix V. Similarly, considerable deviation of class B and smoothened receptors from rhodopsin family at the extracellular part of helix VI did not cause major distance changes from this region to the cytoplasmic end of helix I. These observations suggest that our analysis also provides information on allowable directions along which some helices can deviate without affecting the receptor's functionality. Further accumulation of high-resolution structures could confirm whether the highly diverse backbone organization in the extracellular regions of GPCRs might still be limited by directional restrains that are required to ensure the correct positioning of the cytoplasmic parts of the 7TM bundle.

The most intriguing structural feature of GPCR activation is that a variety of stimuli likely evoke large movement of helix VI while the remaining 6 helices exhibit smaller degree of rearrangement^11^,^18^. For such a mechanism to operate in diverse receptors, we suspect that some common and key intramolecular restraints have been conserved during evolution. In this respect, conservation of the distances from the intracellular side of helix I to the middle/extracellular part of helices III and VI might be an essential requirement.

## Methods

The data processing from the original PDB entries was as follows. Each of the polypeptides in an entry was used to extract the heptahelical bundle, consisting of 200 residues, as described previously^6^. For each polypeptide, C_α_ coordinates were extracted by pdbset in ccp4i^19^. Temperature factor analysis was also performed using these C_α_ files.

In total, 19,900 interatomic distances were calculated and tabulated, using the coordinates of the 200 C_α_ atoms in each bundle for all the chains archived on our web site (www.gses.jp). In cases where two conformers were provided for a residue, frequently W(4.50) in the rhodopsin family receptors, only conformer A was considered because the differences of the C_α_ coordinates between A and B were mostly negligible. Alignment of CRF_1_ receptor in class B, mGluRs in class C, and smoothened receptor was identical to that reported previously^7^,^8^,^9^,^10^ at the most conserved position in each helix. The 7TM bundle of the smoothened receptor (PDB ID: 4JKV) aligned to rhodopsin-like receptors was assumed to lack a residue at the amino terminal of helix IV (4.39). Therefore, in the sets containing the smoothened receptor, the total number of C_α_ pairs was 19,701. Similarly, the 7TM bundle of the class C mGluR1 receptor was assumed to lack two residues at the carboxyl termini of helix II (2.66 and 2.67) and VI (6.59 and 6.60), giving the total number of 18,915 for C_α_ pairs in set 10. Even among the receptors of the rhodopsin family, the possible sequence gaps supposed to occur in a few regions (e.g. the extracellular side of helices II and IV) in some receptors^20^,^21^ were not taken into account, and the BW numbering was serially made in both directions from the *.50 position.

In addition to the criteria for bundle selection for each of the sets, we selected bundles for the minimal sets 9 and 10 as the ones with the highest resolution and lower overall average temperature factor. For instance, chain B of 4AMJ was chosen for the β_1_ adrenergic receptor because 4AMJ has the highest resolution for this receptor and the chain B has lower overall temperature factor for C_α_s than chain A.

In each of the sets, the average, standard deviation, maximum, minimum, and the inverse of coefficient of variation (score) were calculated for the 19,900 or 19,701/18,915 C_α_ pairs. Then sorting was made according to the score. The distribution of the average distances were examined for all 19,900 and the top ranked 1,000 C_α_ pairs of set 1. Two-dimensional plots showing the correlation between the score and the distance were also prepared for set 1.

Molecular dynamics (MD) simulation runs were performed on the delta opioid receptor, having complete inter-helical loops (PDB ID: 4N6H), in a lipid bilayer of 80 × 80 POPC molecules, with NAMD^22^ running on a GPU accelerated PC, using particle-mesh Ewald electrostatics, 10 Å nonbond cut-off, switching function at 9 Å, 12 Å pair list cut-off and 1 fs time step. Briefly, the delta receptor polypeptide (from G36 to G338) having a complete loop connection was inserted into the POPC bilayer, hydrated, neutralized with 0.15 M NaCl by VMD^23^. The whole system was energy minimized for 50 ps and the lipid and the solvent were equilibrated by MD for 0.5 ns under NVT condition (310 K). Then, a 50 ns MD run was performed with no restraints under NPT at 1.0 bar and 310 K and the C_α_-C_α_ distances in the trajectory were analyzed with MD tools implemented in Chimera^24^. All the graphs and the graphics were drawn with Igor Pro (WaveMetrix) and DS visualizer (Accelrys), respectively.

Backbone torsion angles were obtained by DSSP^12^ and the deviations of phi and psi angles among the receptor structures were calculated for each C_α_ position. Then, the values were averaged for each of the 35 sections (5 sections per helix).

## Author Contributions

M.K. performed analysis, T.O. designed research, performed analysis, and wrote the manuscript.

## Supplementary Material

Supplementary Information Supplementary Information

## Figures and Tables

**Table 1:** Conserved pairs within helix III and between helices I and VI

C_α_1	C_α_2	Set 1			Set 2		
Helix III	Helix III	Number of pairs			Number of pairs		
section	section	top1000	all	ratio	top1000	all	ratio
1	2	4	64	0.06	8	64	0.13
1	3	10	32	0.31	13	32	0.41
1	4	34	56	0.61	37	56	0.66
1	5	36	56	0.64	44	56	0.79
2	3	3	32	0.09	7	32	0.22
2	4	42	56	0.75	44	56	0.79
2	5	41	56	0.73	46	56	0.82
3	4	14	28	0.50	17	28	0.61
3	5	21	28	0.75	22	28	0.79
4	4	3	21	0.14	7	21	0.33
4	5	12	49	0.24	16	49	0.33
others		0	83	0.00	2	83	0.02
total		220	561	0.39	263	561	0.47

Helix I	Helix VI	Number of pairs			Number of pairs		
section	section	top1000	all	ratio	top1000	all	ratio
3	1	6	50	0.12	12	50	0.24
3	2	3	25	0.12	4	25	0.16
4	1	31	60	0.52	28	60	0.47
4	2	25	30	0.83	14	30	0.47
5	1	25	50	0.50	16	50	0.32
5	2	20	25	0.80	14	25	0.56
5	3	2	20	0.10	1	20	0.05
others		0	540	0.00	0	540	0.00
total		112	800	0.14	89	800	0.11
