# Supplementary material for: Structural conservation among the rhodopsin-like and other G protein-coupled receptors
Source: Sci Rep. 2015 Mar 17;5:9176. doi: 10.1038/srep09176 (PMC4361874; doi:10.1038/srep09176)
Supplement: Supplementary Information [file srep09176-s1.pdf]

Supplementary Information

Structural conservation among the rhodopsin-like and other G protein-coupled receptors.

Mikitaka Kinoshita, Tetsuji Okada

Fig. S1.

Fig. S2.

Fig. S3.

Table S1.

Table S2.

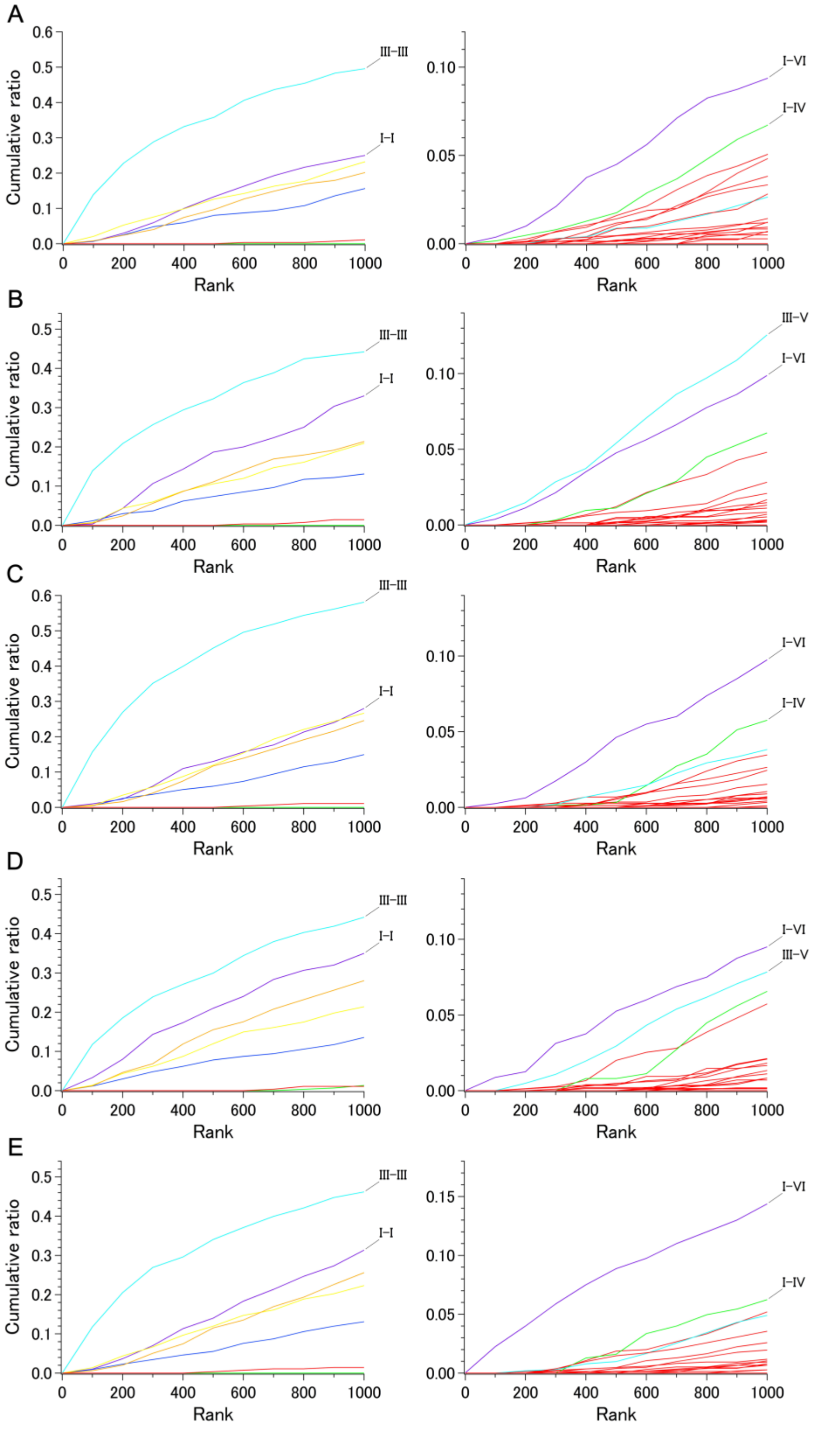

Fig. S1. Cumulative ratio of the number of C_α pairs in the top-ranked 1,000. A. Set 3 (set 2 without rhodopsin). B. Set 4 (set 2 without adrenergic receptors). C. Set 5 (set 2 without adenosine receptors). D. Set 6 (set 2 without chemokine receptors). E. Set 7 (set 2 without opioid receptors). Left: intrahelical pairs, Right: interhelical pairs. Coloring of the plots is the same as in figure 4.

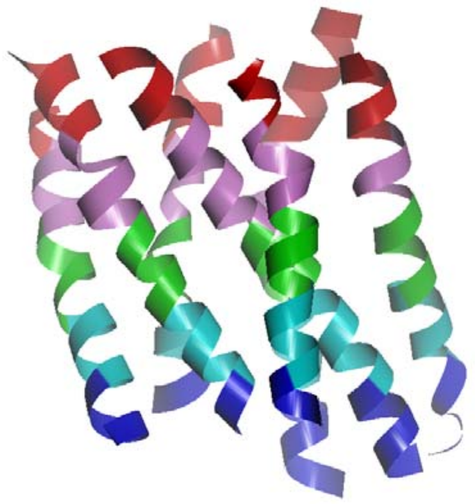

Fig. S2. Horizontal sectioning of the 7TM bundle of GPCRs. Coloring is as follows from the extracellular side: Red, section 1; purple, section 2; green, section 3; cyan, section 4; blue, section 5.

(a)

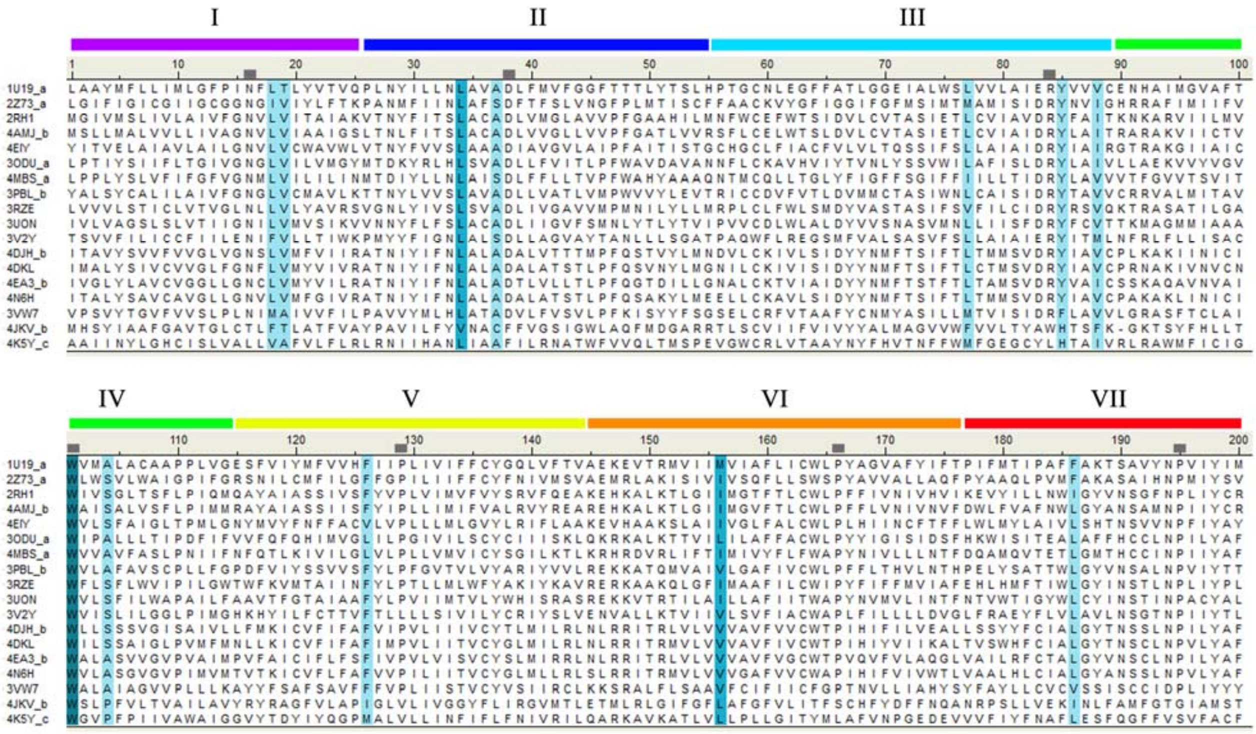
I
II
III
IV
V
VI
VII
1U19_a
2Z73_a
2RH1
4AMJ_b
4EIY
3ODU_a
4MBS_a
3PBL_b
3RZE
3UON
3V2Y
4DJH_b
4DKL
4EA3_b
4N6H
3VW7
4JKV_b
4K5Y_c

(b)

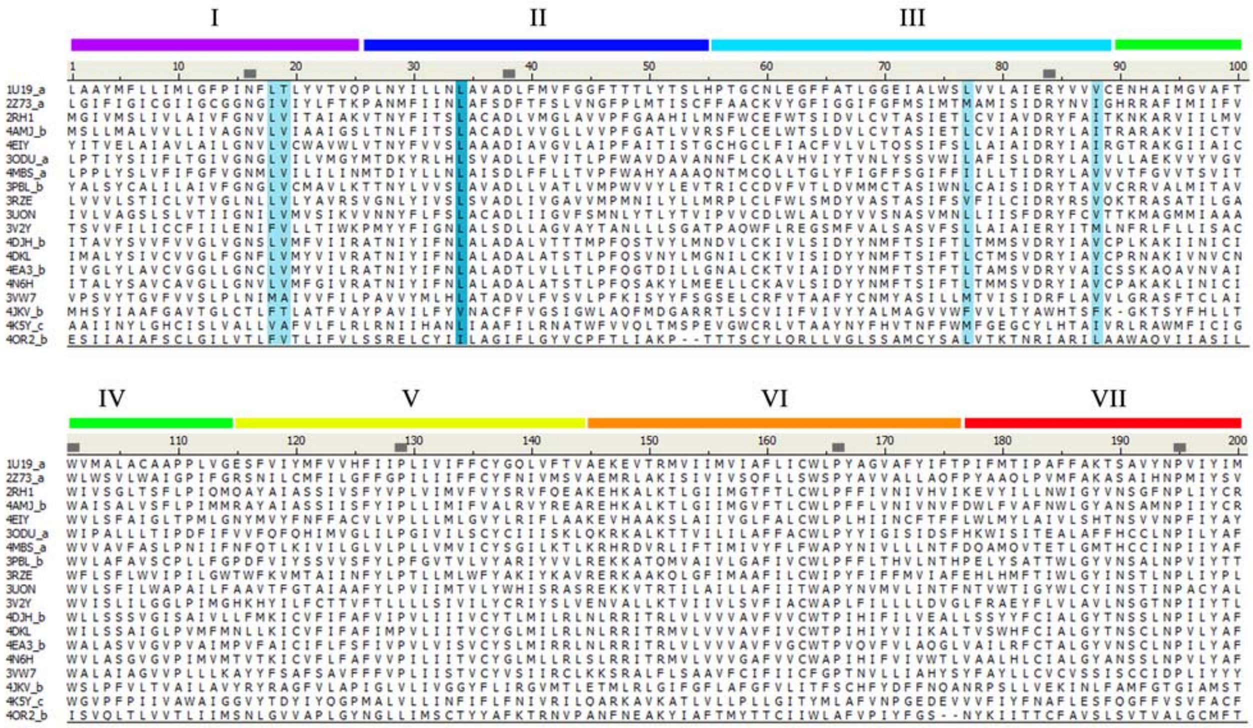
I
II
III
IV
V
VI
VII
1U19_a
2Z73_a
2RH1
4AMJ_b
4EIY
3ODU_a
4MBS_a
3PBL_b
3RZE
3UON
3V2Y
4DJH_b
4DKL
4EA3_b
4N6H
3VW7
4JKV_b
4K5Y_c
4OR2_b

Fig. S3. Sequence alignment of the 7TM regions of eighteen receptors in (a) set 9, and (b) set 10. The conserved positions are colored as follows: dark blue, identical; blue, strong similarity; lightblue, weak similarity, according to PAM250 matrix definition. The small squares on the ruler indicate the *.50 positions of BW numbering for the rhodopsin family.

Table S1. List of chains in the set 0.

receptor	PDB ID					
rhodopsin	1gzm-a	1gzm-b	1u19-a	1u19-b	2j4y-a	2j4y-b	2ped-a
	2ped-b	2z73-a	2z73-b	3ayn-a	3ayn-b		
adrenergic	2rh1	3d4s	3ny8	3ny9	3nya	2vt4-a	2vt4-b
	2vt4-c	2vt4-d	2ycw-a	2ycw-b	2ycy-a	2ycy-b	2ycx-a
	2ycx-b	2ycz-a	2ycz-b	4ami-a	4ami-b	4amj-a	4amj-b
adenosine	3eml	3pwh	3rey	3uza	3uzc	3vg9	3vga
	4eiy						
chemokine	3odu-a	3odu-b	3oe8-a	3oe8-b	3oe8-c		
dopamine	3pbl-a	3pbl-b					
histamine	3rze						
muscarinic	3uon						
sphingosin	3v2w	3v2y					
opioid	4djh-a	4djh-b	4dkl	4ea3-a	4ea3-b	4ej4	
thrombin	3vw7						

Table S2. List of chains in the sets.

Receptor	Type	PDB ID	Set 1	Set 2	Set 3	Set 4	Set 5	Set 6	Set 7	Set 8	Set 9	Set 10	Modification (site)[$]
rhodopsin	bovine	1gzm-a	○	○		○	○	○	○	○			none
rhodopsin	bovine	1gzm-b	○	○		○	○	○	○				none
rhodopsin	bovine	1u19-a	○	○		○	○	○	○	○	○	○	none
rhodopsin	bovine	1u19-b	○	○		○	○	○	○	○			none
rhodopsin	squid	2z73-a	○	○		○	○	○	○	○			none
rhodopsin	squid	2z73-b	○	○		○	○	○	○	○	○	○	none
adrenergic	β_2	2rh1	○	○	○		○	○	○	○	○	○	T4L (ICL3)
adrenergic	β_2	3d4s	○	○	○		○	○	○	○			T4L (ICL3)
adrenergic	β_2	3ny8	○	○	○		○	○	○	○			T4L (ICL3)
adrenergic	β_2	3ny9	○	○	○		○	○	○				T4L (ICL3)
adrenergic	β_2	3nya	○	○	○		○	○	○				T4L (ICL3)
adrenergic	β_1	2vt4-b	○	○	○		○	○	○	○			none
adrenergic	β_1	2vt4-c	○	○	○		○	○	○				none
adrenergic	β_1	2ycy-b	○	○	○		○	○	○				none
adrenergic	β_1	4amj-a	○	○	○		○	○	○	○			none
adrenergic	β_1	4amj-b	○	○	○		○	○	○	○	○	○	none
Adenosine	A_{2A}	3eml	○	○	○	○		○	○	○			T4L (ICL3)
Adenosine	A_{2A}	3uza	○	○	○	○		○	○				None
Adenosine	A_{2A}	3vg9	○	○	○	○		○	○	○			Fab (IC)
Adenosine	A_{2A}	3vga	○	○	○	○		○	○				Fab (IC)
Adenosine	A_{2A}	4eiy	○	○	○	○		○	○	○	○	○	Cyt (ICL3)
chemokine	CXCR4	3odu-a	○	○	○	○	○		○		○	○	T4L (ICL3)

chemokine	CXCR4	3odu-b	○	○	○	○	○		○	○			T4L (ICL3)
chemokine	CXCR4	3oe8-a	○	○	○	○	○		○	○			T4L (ICL3)
chemokine	CXCR4	3oe8-b	○	○	○	○	○		○	○			T4L (ICL3)
chemokine	CXCR4	3oe8-c	○	○	○	○	○		○				T4L (ICL3)
chemokine	CCR5	4mbs-a	○	○	○	○	○		○	○	○	○	Rub (ICL3)
chemokine	CCR5	4mbs-b	○	○	○	○	○		○	○			Rub (ICL3)
Dopamine	D3	3pbl-a	○	○	○	○	○	○	○	○			T4L (ICL3)
Dopamine	D3	3pbl-b	○	○	○	○	○	○	○	○	○	○	T4L (ICL3)
Histamine	H$_1$	3rze	○	○	○	○	○	○	○	○	○	○	T4L (ICL3)
Muscarinic	M2	3uon	○	○	○	○	○	○	○	○	○	○	T4L (ICL3)
sphingosin	S1P$_1$	3v2y	○	○	○	○	○	○	○	○	○	○	T4L (ICL3)
opioid	kappa	4djh-a	○	○	○	○	○	○		○			T4L (ICL3)
opioid	kappa	4djh-b	○	○	○	○	○	○		○	○	○	T4L (ICL3)
opioid	mu	4dkl	○	○	○	○	○	○		○	○	○	T4L (ICL3)
opioid	N/OFQ	4ea3-a	○	○	○	○	○	○		○			Cyt (NT)
opioid	N/OFQ	4ea3-b	○	○	○	○	○	○		○	○	○	Cyt (NT)
opioid	delta	4n6h	○	○	○	○	○	○		○	○	○	Cyt (NT)
thrombin	PAR1	3vw7	○	○	○	○	○	○	○	○	○	○	T4L (ICL3)
smoothened		4jkv-a*		○	○	○	○	○	○	○			Cyt (NT)
smoothened		4jkv-b*		○	○	○	○	○	○	○	○	○	Cyt (NT)
Class B	CRF$_1$	4k5y-c		○	○	○	○	○	○	○	○	○	T4L (ICL2)
Class C	mGluR1	4or2-b[#]										○	Cyt (NT)

* and # indicate that each of the 7TM bundles contains 199 and 196 residues, respectively. [$]Abbreviations: T4L, T4 lysozyme; Cyt, cytochrome; Rub, rubredoxin; ICL, intracellular loop; IC, intracellular surface; NT, amino terminal
